# Continuous Manufacturing of Microfluidic Fibers Embedded with Ordered Microparticles via Ionic Gelation

**DOI:** 10.1021/acsaenm.2c00060

**Published:** 2022-09-23

**Authors:** Antonio Maisto, Daniel McDowall, Dave J. Adams, Francesco Del Giudice

**Affiliations:** † Department of Chemical Engineering, School of Engineering and Applied Science, Faculty of Science and Engineering, Swansea SA1 8AJ, U.K.; ‡ School of Chemistry, University of Glasgow, Glasgow G12 8QQ, U.K.

**Keywords:** particle ordering, viscoelasticity, biomaterials, fibers, non-Newtonian fluids, gelation

## Abstract

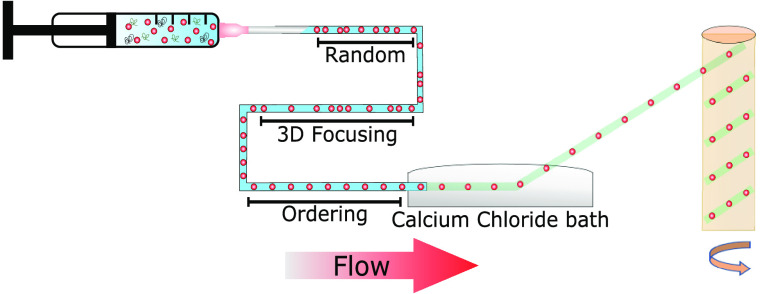

Fibers loaded with either particles or cells are widely employed across a variety of fields, including material science, tissue engineering, and pharmaceutical research. However, the concentration of such objects along the fiber length remains stochastic, thus resulting in fibers having heterogeneous properties along their length. We here introduce a new class of material featuring fibers loaded with “equally spaced” microparticles. The fibers were obtained thanks to the combination between the recently discovered viscoelastic particle ordering phenomenon and the well-established process of fiber synthesis via ex situ ionic gelation. We employed a simple experimental apparatus made of a syringe pump connected to a 100 μm tube ending in a calcium chloride bath. The liquid forming the fiber was an aqueous solution of hyaluronic acid and sodium alginate. We studied the effect of volumetric flow rate, sodium alginate concentration, and spinning speed on the fiber diameter and the particle-spacing in the fiber. We also discussed the advantages of this type of fiber over the existing ones and suggested potential applications across several fields.

## Introduction

Fibers are long flexible materials having a diameter on the order of 10s of micrometers and a length of at least 1000 times their diameter.^1^ Fibers have seen an exponential growth in their use thanks to the advancement of micro/nanofabrication technologies.^1−3^ Fibers have been successfully used across a variety of fields, including electronics, cosmetics, pharmaceutical research, biomedical engineering, and tissue engineering.^4^ For instance, fibers have been used for cell regulation studies,^5^ for the manufacturing of muscle-like tissues,^6^ or for drug-release studies.^7,8^

Microfluidics has played a pivotal role in facilitating production of fibers with controllable and homogeneous properties, compared to the most classic bulk techniques.^3^ In fact, microfluidic technologies deal with the manipulation of fluids down to the nanoliter scale,^9^ leading to unprecedented control over physical properties and fabrication methodologies, thus resulting in fibers manufactured using methodologies that are highly reproducible.^3,10^

The current state-of the art^4,10^ includes fibers having different shapes but made of a single material,^11−14^ fibers loaded with cells to enable formation of tissues,^6,15,16^ fibers loaded with nanoparticles,^17^ or nonspherical nanometer-sized objects.^18^ The main limitation faced by fibers containing either cells or particles is the fact that their distribution within the fiber is stochastic, as it is not possible to insert them uniformly. Such limitations can result in a series of drawbacks, including the fact that fibers may present heterogeneous properties along their length (in the case of particles) or that intercellular interactions along the fiber are not uniform, meaning that the resulting tissue may present a heterogeneous configuration. Clearly, the possibility of controlling the distribution of objects within the fiber may address such drawbacks while also prompting new research directions in the development of new materials. The most successful attempts were made by combining the formation of droplets in microfluidic devices and the ex situ ionic gelation process.^19^ Specifically, spherical^19^ and nonspherical^20^ droplets were formed at a microfluidic junction where two nonmiscible liquids met;^21^ the formed droplets were then subsequently surrounded by an alginate solution that, upon contact with a calcium chloride bath, led to the formation of rigid fiber-containing droplets (i.e., via ex situ ionic gelation). Since the frequency of droplet formation is constant and controlled by the volumetric flow rate of the two nonmiscible liquid streams, a variety of fibers with equally spaced droplets could be formed. Some variations to this approach have led to the synthesis of micromotors,^20^ microactuators,^22^ and biomimetic microscale materials.^23^ In all these cases, however, the material in the fiber was relatively soft, without considering more rigid objects such as particles or cells. Some attempts have recently been made to equally space cells by encapsulating them in equally spaced droplets before the gelation process took place.^24^ However, the encapsulation process is also stochastic, with the encapsulation efficiency controlled by the Poisson statistics,^25^ which is around 30% for single encapsulation. The possibility of manipulating the distance between rigid objects in fibers has, so far, remained out of reach.

We here introduce a new class of material featuring fibers loaded with equally spaced microparticles. This result is achieved by combining the recently discovered viscoelastic particle ordering phenomenon in microfluidic devices^26^ with the well-established ex situ chemical gelation principle.^27,28^ We employ a simple experimental apparatus made of a syringe pump connected to a tube having an internal diameter of 100 μm exiting in a calcium chloride bath. The liquid suspending the particles is an aqueous solution containing hyaluronic acid and sodium alginate. The flow properties of the suspending liquid coupled to the flow conditions in the tube and to the ex situ gelation mechanism lead to the continuous manufacturing of fibers with equally spaced particles.

We first provide some information regarding the working principle behind the manufacturing of this class of materials, before presenting the results of our experimental campaign followed by a discussion related to the advantages of such fibers over the state of the art, and their potential use across different fields.

## Working Principle

**Figure 1:**
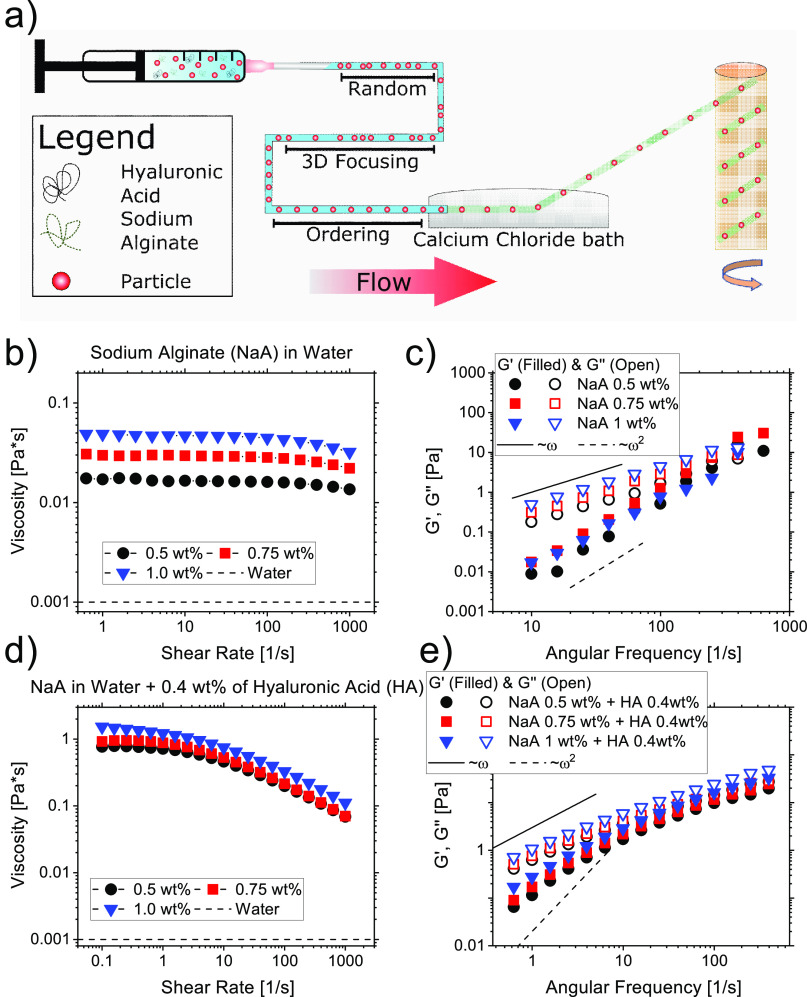
(a) Schematics of the working principle. Particles suspended in an aqueous sodium alginate (NaA) and hyaluronic acid (HA) solution flow in to the microfluidic tube. Particles first align on the tube centerline because of the viscoelastic forces acting on them^37−39^ and then self-ordered thanks to the viscoelasticity-mediated hydrodynamic interactions.^26,36^ (b) Viscosity curve and (c) frequency sweep experiments for several aqueous solutions of NaA. (d,e) Same as in (b,c), respectively, for NaA solutions with the addition of 0.4 wt % of HA. The slopes in (c,e) are the theoretical predictions for the terminal viscoelastic region.^34^

The formation of fibers with equally spaced particles is driven by the synergy between two phenomena, namely the viscoelasticity-induced particle ordering^26,29^ and the formation of fibers thanks to ex situ chemical gelation.^27,28^ While the formation of fibers via ex situ gelation is a well-established and understood process,^30^ the same is not true for the particle ordering.^31^ In accordance with the existing studies on the subject, the formation of strings of equally spaced particle structures (i.e., particle ordering) occurs when the liquid suspending the particles presents shear-thinning properties,^26,32,33^ meaning that the shear-viscosity decreases when increasing the flow rate in the microchannel.^34^ The particle ordering is a two-step phenomenon.^26,33,35,36^ Particles suspended in the syringe are randomly distributed along the cross-section, meaning that, upon entering the microchannel, their distribution remains random (Figure 1a). While flowing in the microchannel, the viscoelasticity of the suspending fluid exerts a net force on the flowing particles and pushes them toward the channel centerline, leading to the so-called 3D focusing,^37^ meaning that particles are “focused” on a single streamline. For fluids presenting shear-thinning properties (such as those employed in this study), the 3D focusing is only possible when the confinement ratio β = *d*/*D*, where *d* is the particle diameter and *D* is the channel diameter, is such that^38,39^ β ≥ 0.2. The transition from simple 3D focusing (where particles are all aligned but their mutual distance is not constant) to particle ordering (where particles are equally spaced) occurs thanks to the hydrodynamic interactions between consecutive particles^35,40^ (Figure 1a). The transition from alignment to ordering is generally slow, and it requires long channel lengths to develop.^36,40^ More specifically, it has been shown^36,40^ via numerical simulations and experiments that the ratio between the channel length *L* and the tube diameter *D* can be as large as *L*/*D* ≥ 2500 in order to observe a distribution of equally spaced particles for a confinement ratio β = 0.2. The dynamics of particle ordering is instead quicker when increasing the confinement ratio, meaning that smaller values of *L*/*D* are required to observe particle ordering at larger β values. It is currently not possible to be more quantitative regarding specific values of the geometrical parameters, as the ordering phenomenon remains not fully understood. The confinement ratio should be β = *d*/*D* ≥ 0.2, otherwise particles suspended in the shear-thinning suspending liquid would not align at the channel centerline but rather near the channel walls.^41−43^ The requirement for particles to self-assemble in ordered structures before the fiber could be formed led to the development of an experimental apparatus that could then promote the continuous formation of fibers containing equally spaced particles.

A schematics for the experimental setup is reported in Figure 1a. Particles having a diameter *d* = 20 μm are first added to an aqueous shear-thinning viscoelastic solution made of hyaluronic acid at 0.4 wt % and sodium alginate; the sodium alginate is required for the formation of the fiber.^21,30,44^ The resulting suspension is then loaded into a syringe connected to a 60 mm long cylindrical tube with internal diameter of 100 μm. While flowing into the channel, particles first align and then form equally spaced structures at the channel centerline (Figure 1a). At the end of the channel, the suspension enters a plastic Petri dish containing an aqueous solution of calcium chloride, where calcium chloride ions diffuse within the fiber containing sodium alginate, thus leading to the ex situ gelation of the fiber, which is then collected using an external rotating motor.

## Materials and Methods

### Sample Preparation and Characterization

An aqueous stock solution of sodium alginate (NaA, Sigma-Aldrich) at 5 wt % was prepared by adding the NaA powder to the deionized water on a stirrer for 12 h to allow full dissolution. The deionized water presented a conductivity of 18.2 mΩ cm at 25 °C, and it was sourced from tap water filtered in a ultrapurification unit available in the laboratory. A stock solution of hyaluronic acid (HA, Sigma-Aldrich) with molecular weight *M*_*w*_ = 1.5–1.8 MDa at 0.5 wt % and a stock solution of calcium chloride (Sigma-Aldrich) at 1.5 wt % were prepared in the same manner. The NaA solutions at 0.5, 0.75, and 1 wt % were prepared by direct dilution of the NaA 5 wt % stock. The solutions containing both NaA at different concentrations and HA at 0.4 wt % were prepared by diluting the NaA 5 wt % stock with the HA 0.5 wt % stock to the desired NaA final concentration, thus leading to a reduction in the HA concentration down to 0.4 wt %. The concentration of hyaluronic acid was kept constant to 0.4 wt %.

The rheological measurements to characterize the solutions were carried out using a conventional stress-controlled rheometer (TA 2000ex) equipped with a parallel plate configuration (aluminum plate with diameter of 60 mm), constant gap between plates of 400 μm, and constant temperature of 20 °C. All the NaA solutions displayed a viscosity that was nearly constant over the whole range of shear-rate investigated (Figure 1b), with a minor shear-thinning observed above a shear rate of around 800 s^–1^. We also carried out small amplitude oscillatory shear (SAOS) measurements to quantify the viscoelastic properties of the solutions (Figure 1c). In such measurements, the storage modulus *G*′ and the loss modulus *G*″ that quantify the elastic and the viscous response, respectively, of the material are evaluated at different oscillatory angular frequencies ω. Regardless of the polymer concentration, the solutions displayed marked viscoelastic properties with data at low angular frequency values (i.e., the so-called terminal region) scaling with slopes 1 and 2, as predicted by the theory.^34^ The same rheological analysis was repeated on the solutions containing NaA at different concentrations and HA at 0.4 wt % (Figure 1d,e). The solutions containing HA displayed a more marked shear-thinning compared to those without HA (Figure 1b), meaning that they are more suitable to observe the particle-ordering phenomenon.^26^ The zero-shear viscosity increased by around 1 order of magnitude compared to the solutions without HA, while the elastic response remained relatively similar (Figure 1e).

Rigid neutral polystyrene particles (polysciences) having diameter *d* = 20 ± 2 μm were added to the solutions in order to achieve a final bulk particle concentration of 0.4 wt %. To avoid contamination, the fluid suspending the stock particles was removed via centrifugation and then replaced with the solutions containing NaA and HA. The resulting system was put on a vortex mixer (Fisherbrand) to mix the suspension. Even though the polystyrene particles were not density-matched to the solution, particle settling was negligible in our experiments because of the large value of the zero-shear viscosity for our solutions (Figure 1d). By using the Stokes law, we estimated a settling velocity of 1.09 × 10^–2^ μm/s, leading to a settling time to cover the syringe radius (2.25 mm) of around 57 h, far beyond the duration of the experiments.

### Experimental Apparatus and Methodology

An inverted microscope (Zeiss Axiovert A1) was used to analyze particle flow in a 60 mm long commercial tube with internal diameter of 0.10 mm (Dolomite Microfluidics) with a circular section and external diameter of 1.6 mm. The tube was directly attached to a 1 mm glass syringe (Hamilton, luer ending) via a 1/4″–28″ threaded fitting (Dolomite Microfluidics, equivalent to an M6 × 0.75 metric fine) connected to a female-to-female luer lock (Dolomite Microfluidics). Videos of flowing particles were captured using a fast camera (Photron, FASTCAM Mini UX50) at 2000 fps or 3200 fps depending on the polymer concentration and the imposed volumetric flow rate. The suspensions were pumped in the Petri dish containing the 1.5 wt % calcium chloride bath at different volumetric flow rates *Q* in the range of 10–80 μL/min through a syringe pump (KD scientific). A homemade motor was employed to spin the fiber out from the tube at different velocities. It was built by using a power supply chip with different voltages to change properly the angular velocity of the motor, a common breadboard and a 3–12 V electric motor. For the purposes of this work, three angular velocity parameters were defined, called Ω_0_, Ω_1_, and Ω_2_, with Ω_0_ = 0 rpm (rotations per minute), Ω_1_ = 110 rpm, and Ω_2_ = 167 rpm.

The experimental videos were then analyzed using a in-house MATLAB subroutine to derive the distance between consecutive particles. The distance was measured from center to center and divided by the particle diameter to obtain a normalized distance between particles, S* = *s*/*d*, where *s* is the distance center-to-center and *d* is the particle diameter. Interparticle distance histograms were then obtained with a binning size equal to 1 and boundary ends were set to 0 and 64.5 which is the total length of the observation window.

## Experimental Results

### Manufacturing of Fibers with Embedded Ordered Particles

**Figure 2:**
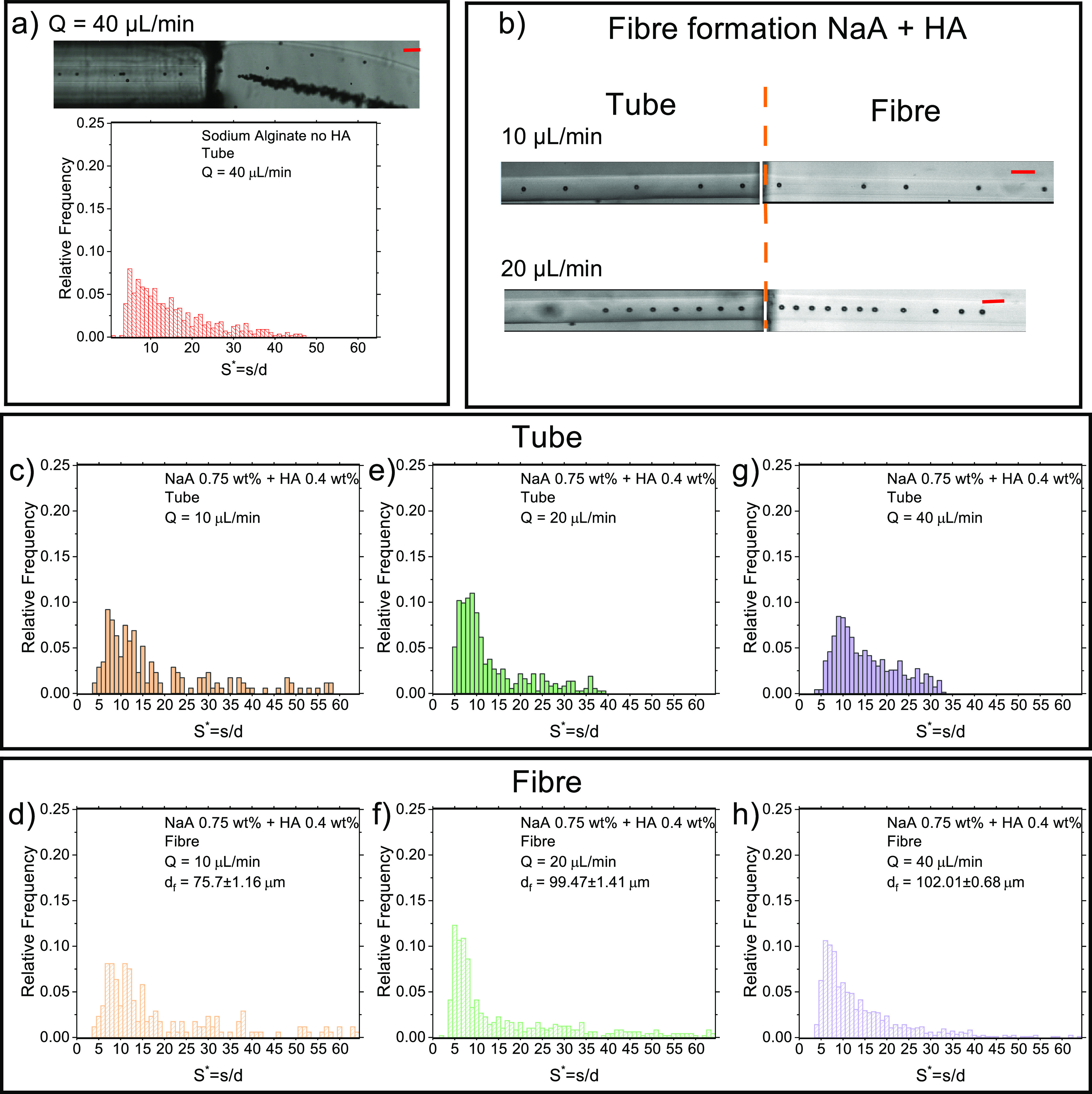
Manufacturing of fibers with embedded ordered particles. (a) Particles suspended in sodium alginate (NaA) without hyaluronic acid (HA) focus on the tube centerline, but they do not self-order, as demonstrated by the lack of a peak in S* = *s*/*d*, where *s* is the distance between consecutive particles and *d* is the particle diameter. Scale bar is 100 μm. (b) Experimental snapshots displaying particle self-ordering within the tube (left) and within the fiber (right); the suspending liquid is an aqueous NaA 0.75 wt % with the addition of 0.4 wt % of HA. Scale bar is 100 μm. (c–h) Distributions of S* in the tube and in the fiber for different values of the volumetric flow rate. The spinning speed is Ω_1_ = 110 rpm.

We first performed experiments to check whether the aqueous sodium alginate (NaA) solutions without the hyaluronic acid (HA) could lead to the formation of fibers with embedded equally spaced particles (Figure 2a). Particles suspended in a NaA 1 wt % solution flowing in the tube, arrived aligned on the centerline but with a normalized distance S* distribution that did not present any clear peak (histograms in Figure 2a and Video S1), meaning that particles were not equally spaced. Upon entering the calcium chloride bath, the fiber was formed instantaneously; however, particles collapsed on to each other to form a long disordered string (snapshot in Figure 2a and Video S1). Within the tube, we also observed the presence of long strings of attached particles, suggesting that particles in close proximity to each other experienced attractive hydrodynamic interactions, which are common when the fluid is elastic but presents a near-constant viscosity,^26,32,33^ which is the case of the NaA solution without HA (Figure 1b). A similar behavior was also observed for other imposed volumetric flow rate values (data not shown).

The addition of 0.4 wt % HA to a NaA 0.75 wt % solutions resulted in the formation of fibers with equally spaced structures (Figure 2b, Video S2). Indeed, the suspending liquid was now shear-thinning (Figure 1d) meaning that consecutive particles would mainly experience repulsive hydrodynamic interactions (in contrast with the attractive hydrodynamic interactions experienced for near-constant viscosity liquids), as previously reported in the literature.^26,32,33^ We observed a string of equally spaced particles in the tube before the gelation process took place (Figure 2b) at both 10 and 20 μL/min, with the fibers that were subsequently formed upon contact with the calcium chloride bath, meaning that the presence of HA in the suspending liquid at such relatively small concentrations (0.4 wt %) did not affect the gelation chemistry significantly. For all the experiments, a constant spinning speed Ω_1_ = 110 rpm was applied to remove the fiber from the bath in a continuous way. We also quantified the effect of the volumetric flow rate on the distance between particles observed before and after the gelation process (Figure 2c–h). At *Q* = 10 μL/min, particles flowing in the tube presented a relatively broad distribution of normalized distances in the range of 5 < S* < 20 (Figure 2c); upon gelation, we did not observe significant changes in the particle distribution in the fiber (Figure 2d). An increase of the volumetric flow rate to *Q* = 20 μL/min, led to an improvement of the particle ordering, with a distribution of normalized distances in the range of 5 < S* < 10, meaning that more particles were equally spaced compared to the case at *Q* = 10 μL/min. This result is in agreement with recent findings on viscoelastic particle ordering,^26,40^ where it was observed that, for a given channel length, larger volumetric flow rate values led to more particles being equally spaced. The S* distribution at *Q* = 10 μL/min in the tube was slightly more narrow than the one in the fiber (Figure 2c,d), as the fiber spinning process resulted in a fiber with a diameter of around 76 μm, meaning that particles slightly accelerated in the fiber because of the reduction in the cross-section compared to the 100 μm tube. For volumetric flow rate values of 20 (Figure 2e,f) and 40 μL/min (Figure 2g,h), the distribution remained substantially unaltered because the cross-section of the fiber was nearly identical to the 100 μm tube. It is important to mention that any change in the S* distribution between the tube and the fiber is strictly linked to the relation between the imposed volumetric flow rate and the constant angular velocity of the rotating motor to spin–out the fibers. The larger the volumetric flow rate of the fluid, the larger will be the spinning speed required to keep the formed fiber at a constant diameter equal to the tube diameter. In our investigation, we instead kept the spinning speed constant because our homemade motor did not allow a continuous change in the spinning speed. Furthermore, to present the data consistently, we kept the spinning speed at a constant value. More details regarding the results at different speed values are discussed later. It is also important to notice that the volumetric flow rate did not have a strong impact on the location of the peak in the normalized distributions, in agreement with the fact that the distance between particles is determined by the particle diameters, the channel diameter, and the particle concentration^26^ but not by the volumetric flow rate. Large values of the volumetric flow rate are only expected to lead to more particles equally spaced in ordered structures over a shorter channel length.^26^ It is important to note that the particle distributions in Figure 2 were also affected by the well-established phenomenon of particle concentration fluctuations,^40^ meaning that, even if the particles were homogeneously distributed in the syringe, the presence of contraction areas between the syringe and the tube caused fluctuations in the particle concentration.

In summary, we here demonstrated the continuous formation of fibers with embedded equally spaced microparticles. The HA was clearly required to observe the ordering phenomenon. The normalized particle distance distribution did not change significantly with the volumetric flow rate, as previously observed for experiments on viscoelastic ordering.^26^ We also observed that the fiber could be formed instantaneously in the bath, meaning that neither the presence of the particles nor the HA interfered with the chemical gelation.

### Effect of Sodium Alginate Concentration

After demonstrating the continuous formation of fibers embedded with ordered particles, we investigated the effect of the NaA concentration on the fiber formation, while keeping the HA concentration constant to 0.4 wt % and the spinning speed constant at Ω_1_ = 110 rpm. An increase of the NaA concentration in solution resulted in a slightly more enhanced shear-thinning behavior (Figure 1d) and in an increase of the elasticity (Figure 1e). While the viscosity data in the shear-thinning region followed more or less the same slope with increasing the shear rate (Figure 1d), the elasticity of the solution, quantified via the storage modulus *G*′ increased by around 4 times between NaA 0.5 wt % and NaA 1 wt % (Figure 1e). An increase of the elasticity of the solution means that, for a constant volumetric flow rate, more particles could potentially be ordered compared to smaller flow rate values.^26,33^

**Figure 3:**
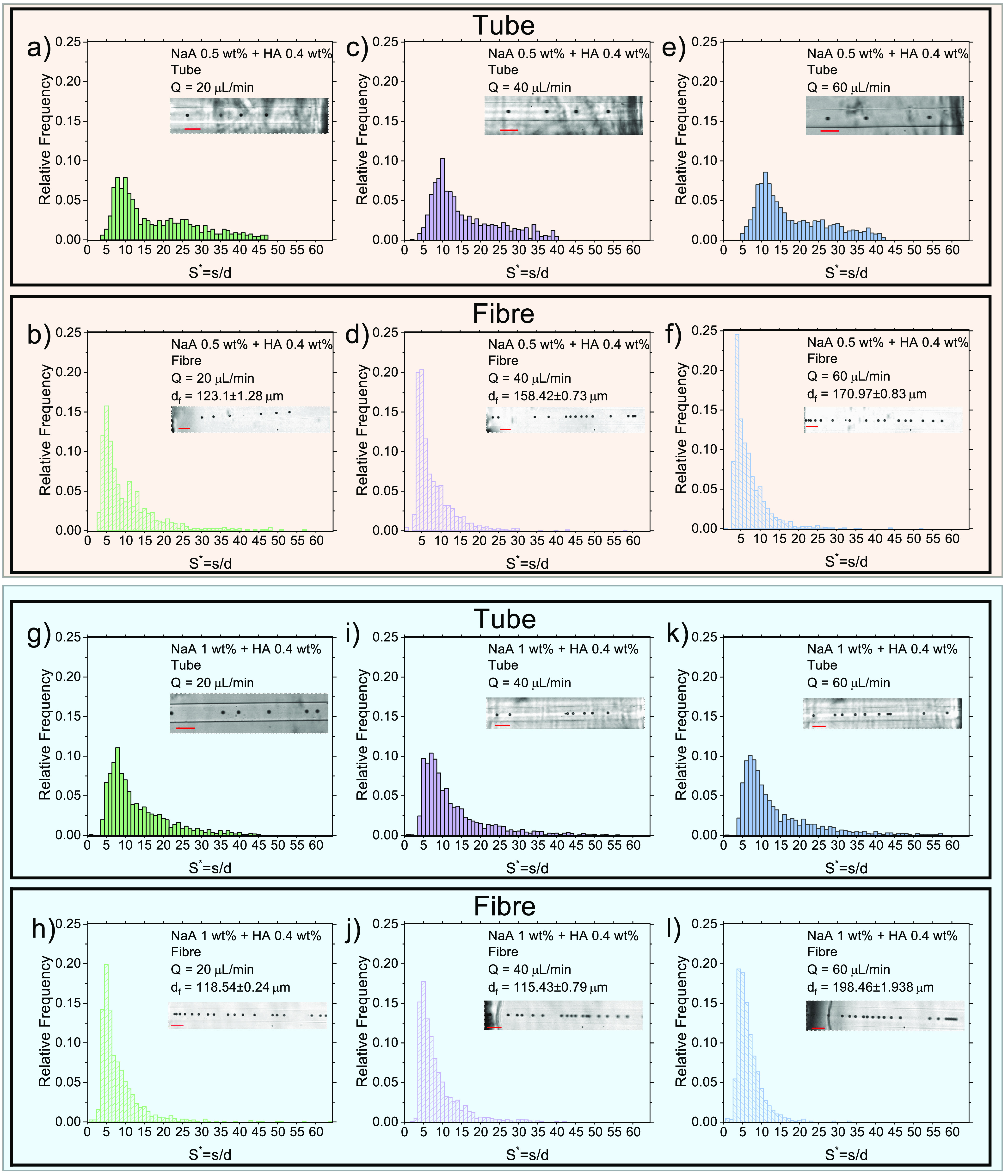
Effect of sodium alginate (NaA) concentration on the particle distribution in the tube and in the fiber. (a–l) Distributions of S* in the tube and in the fiber for different values of the volumetric flow rate *Q* and of the mass concentration of NaA, while keeping HA constant to 0.4 wt %. The spinning speed is Ω_1_ = 110 rpm. Experimental snapshots are taken from the recorded videos. Particle size is 20 μm in all the videos. Different background shades correspond to different NaA concentrations. Scale bar is 100 μm.

We performed the same experiments presented in the previous paragraph for a NaA concentration of 0.5 wt % and for a constant HA concentration of 0.4 wt % (Figure 3a-f). For *Q* = 20 μL/min (Figure 3a,b), we observed distributions very similar to the 0.75 wt % (Figure 2e,f), with a peak in the distribution of particles in tube in the range of 5 < S* < 15, and with a tiny increase in the fraction of ordered particles in the fiber, exactly as for the case of NaA 0.75 wt %. The S* distribution in the fiber was less broad compared to the one in the tube, as the fiber diameter increased to around 123 μm, larger than the 100 μm tube, meaning that particles decelerated and found themselves at a shorter distance between each other. We repeated the experiments for the volumetric flow rate values of *Q* = 40 μL/min (Figure 3c,d) and *Q* = 60 μL/min (Figure 3e,f), observing a trend similar to the one observed for particles suspended in NaA 0.75 wt %. An increase of the NaA concentration up to 1 wt % resulted in a slight increase in the fraction of particles ordered in tube and in the fiber (Figure 3g–l). In agreement with the experiments presented previously, we observed an increase of ordered particles in the fiber, again as a consequence of the fact that particles were slightly closer to each other in the fiber compared to the tube, thus leading to enhanced hydrodynamic interactions with more particles for the same fiber length.

Taken together, these results demonstrated that fibers are relatively stable in the narrow range of NaA concentrations investigated. The distribution of particles in the tube and the fiber remained substantially unaltered in terms of peak location, but more particles were ordered at larger NaA concentration, in agreement with previous studies on particle ordering.^26,33^

### Effect of Spinning Speed

**Figure 4:**
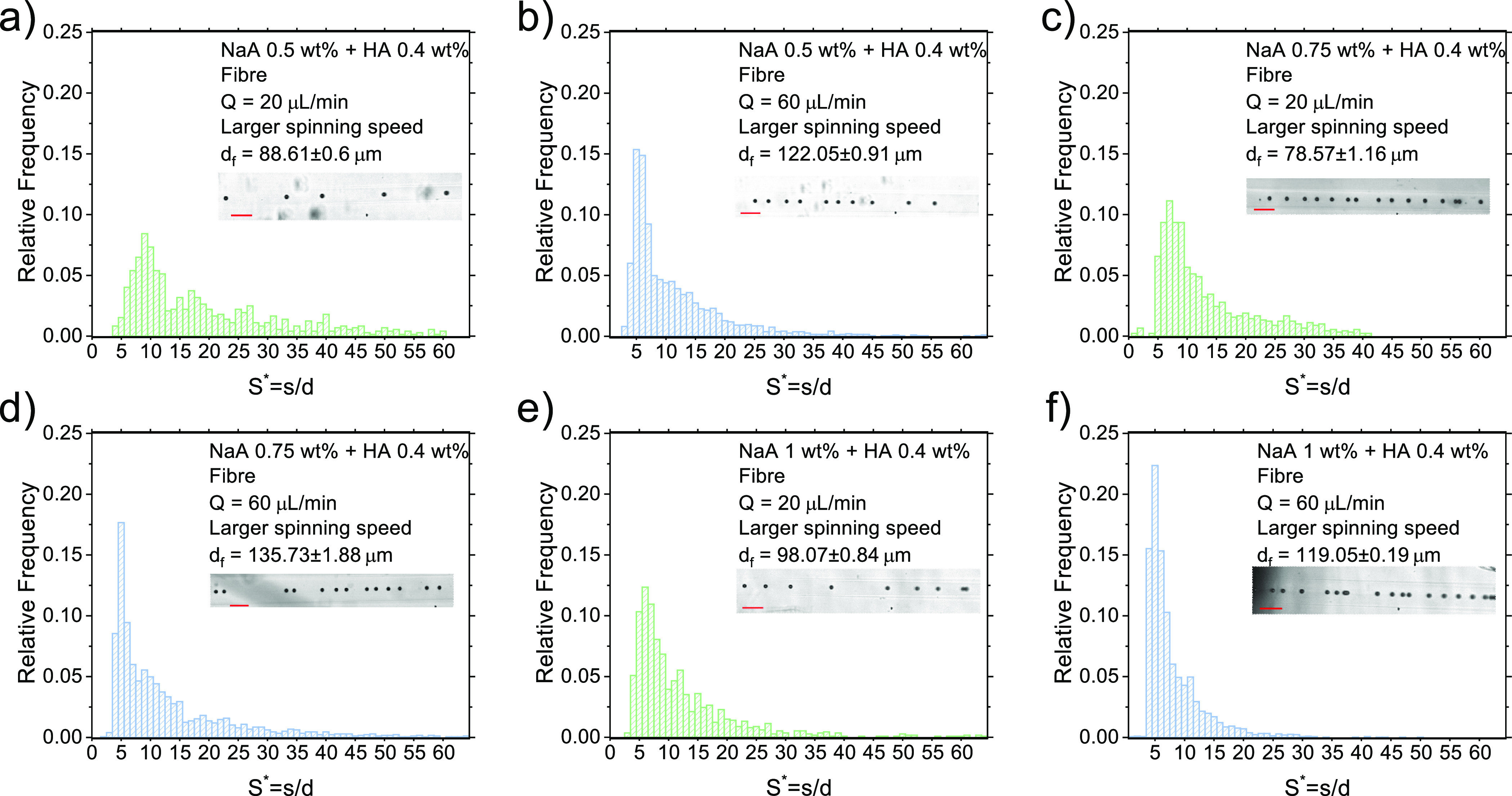
Effect of spinning speed on the particle distribution in the tube and in the fiber. (a–f) Distributions of S* in the fiber for different values of the volumetric flow rate *Q* and of the mass concentration of NaA, while keeping HA constant to 0.4 wt %. Here, the spinning velocity to collect the fiber is larger than the one employed for the results of Figure 2 and Figure 3, being Ω_2_ = 167 rpm. Experimental snapshots are taken from the recorded videos. Particle size is 20 μm in all the videos. Scale bar is 100 μm.

We also performed an experimental campaign to investigate how the spinning speed could impact the formation and the properties of the formed fibers. Specifically, we performed experiments of fiber formation when (i) the motor was not spinning out the fiber (i.e., at Ω_0_ = 0 rpm), (ii) the motor was spinning at angular velocity Ω_1_ = 110 rpm (i.e., the results presented before), and (iii) the motor was spinning at an angular velocity Ω_2_ = 167 rpm (Figure 4). We repeated the experimental campaign reported before for the solutions containing HA at 0.4 wt % and NaA at 0.5, 0.75, and 1 wt %, for volumetric flow rate values of 20 and 40 μL/min, but for different spinning speed values. When the motor was not spinning (Ω_0_ = 0 rpm), the fiber still formed; however, the diameter of the fiber was significantly larger than the one of the tube, and the formed fiber was fluctuating in the Petri dish, preventing any analysis of the S* distribution (Video S3). When the motor was not spinning, the fibers always displayed a diameter significantly larger than the tube with particles forming several aggregates because of the strong deceleration experienced in the fiber (Video S3): this phenomenon was more obvious at larger volumetric flow rate values (Video S4 and Video S5).

The situation was different when the spinning speed was set to Ω_2_ = 167 rpm (Figure 4). Regardless of the minor quantitative differences observed for each experiment, we noticed that the fiber diameter was always smaller than the one of the fiber derived when the spinning velocity was set to Ω_1_ = 110 rpm (compare Figure 2, Figure 3, and Figure 4), exactly as expected. For instance, for NaA 0.75 wt % at *Q* = 20 μL/min, we observed a decreasing in the fiber diameter from around 100 μm for spinning speed Ω_1_ (Figure 2f) to around 78 μm (Figure 4c and Video S6). These results confirm that volumetric flow rate and spinning speed can actually be controlled to manufacture fibers having different diameters. According to the existing literature,^26,36,40^ the particle bulk concentration (which was always kept constant in our analysis) can instead be tuned to modify the particle–particle distance in the fiber.

A summary of the values of the measured fiber diameter for all the experiments performed is shown in Table 1.

**Table 1:** Fiber Diameter As a Function of the Spinning Speed and the Sodium Alginate Concentration^a^

NaA 0.5 wt % and HA 0.4 wt %					
	10 μL/min	20 μL/min	40 μL/min	60 μL/min	80 μL/min
Ω_0_	160 ± 0.6 μm	151 ± 0.1 μm	193 ± 1.0 μm	199 ± 1.0 μm	230 ± 1.0 μm
Ω_1_	89 ± 2.0 μm	123 ± 1.0 μm	158 ± 1.0 μm	171 ± 1.0 μm	180 ± 1.0 μm
Ω_2_	73 ± 0.8 μm	89 ± 1.0 μm	122 ± 0.9 μm	122 ± 0.9 μm	162 ± 0.8 μm

NaA 0.75 wt % and HA 0.4 wt %					
Ω_0_	170 ± 0.7 μm	165 ± 1.4 μm	143 ± 0.6 μm	179 ± 0.6 μm	214 ± 4 μm
Ω_1_	76 ± 1.0 μm	99 ± 1.4 μm	102 ± 0.7 μm	198 ± 2.0 μm	211 ± 2.0 μm
Ω_2_	65 ± 1.2 μm	79 ± 1.2 μm	83 ± 0.7 μm	136 ± 3.9 μm	118 ± 1.0 μm

NaA 1 wt % and HA 0.4 wt %					
Ω_0_	152 ± 0.1 μm	156 ± 0.5 μm	166 ± 0.7 μm	188 ± 0.8 μm	219 ± 1.0 μm
Ω_1_	85 ± 0.9 μm	119 ± 0.2 μm	115 ± 0.8 μm	165 ± 0.8 μm	145 ± 0.6 μm
Ω_2_	56 ± 0.04 μm	98 ± 0.8 μm	112 ± 0.6 μm	119 ± 0.2 μm	81 ± 0.1 μm

a The concentration of hyaluronic acid was kept constant at 0.4 wt %. The angular velocity Ω_0_ = 0 rpm means that the motor was not employed to spin the fiber. The spinning velocity Ω_1_ = 110 rpm is smaller than Ω_2_ = 167 rpm.

## Discussion

We have here manufactured a new class of materials made of fibers containing equally spaced structures. The key innovation is the exploitation of the viscoelastic particle ordering phenomenon recently discovered^26^ within the framework of fiber synthesis.^4,10^ The whole experimental apparatus is also very simple, and it features an on-chip formation of particle structures followed by the ionic gelation of fiber, similarly to the wet-spinning fiber fabrication procedure,^4^ which requires minimal equipment compared to other procedures such as 3D bioprinting and electrospinning. Needless to say, fibers with uniformly loaded particles could also be produced using either 3D bioprinting or electrospinning or any other fiber fabrication technique, with the only requirement that the conditions for the particles to equally space within the tube are met. Clearly, this offers a high level of versatility to the fibers introduced here, with our results calling for new studies in this direction. By modulating the properties of both the suspending liquids and the particles, new materials can be introduced relatively easily. For instance, strings of attached particles in fibers can be manufactured thanks to the attractive nature of the viscoelasticity-mediated hydrodynamic interactions when the suspending liquid presents a near constant-viscosity^26^ and when the particle concentration is significantly large.^33,40^ In accordance with the recent findings on viscoelastic ordering,^26,33,40^ an increase in the bulk particle concentration such that particles are not too close to each other result in particles being equally spaced at a shorter distance compared with the case of lower bulk concentrations. Of course, the particle concentration needs to be sufficient for the particle to hydrodynamically interact, meaning that too small particle concentrations cannot lead to equally spaced structures.^26^ The chemistry of the suspending liquid is instead critical to allow the formation of the fiber, while also preventing unwanted interactions between suspending liquid and particles.

The fibers introduced here are very different from the ones currently available from the state of the art,^4,10^ and they carry significant potential for the generation of new versatile materials. The majority of previously published works focused on the manufacturing of “unloaded” fibers, including hollow fibers,^11^ fibers having different complex cross sections (e.g., triaxial,^12^ E-shaped^13^), or helical fibers.^14,21^ While there is no question regarding the importance of such fibers, we stress the fact that the addition of particles result in an increased layer of complexity in the nature of the resulting fibers, opening new avenues to be explored. Some works have introduced cells within the fibers to manufacture tissues.^15,44^ For instance, neuron cells have been added to the fibers so that they could form links with nearby cells and form a network in the confined fiber environments.^15^ Other studies have instead focused on having cells cultured within the fiber in order to replicate the behavior observed in tissues.^44^ Nanoparticles have also been added to the fibers to improve drug delivery applications,^17^ and graphene oxide sheets have been introduced in fibers to create a multiphase and multifunctional fiber.^18^ However, the objects added to the fibers were always in a random distribution rather than having a constant distance between each other. For nanometer-scale objects, this is not a major problem as they will fill in the cross-section of the fiber without significant heterogeneity. However, when moving to micrometer-sized objects in fibers, the same behavior cannot be expected because of the large hindrance of particles within micrometer-sized fibers, thus resulting in fibers having heterogeneous properties along their length; fibers loaded with equally spaced particles represent a suitable alternative to address this challenge. By replacing particles with cells, the fibers introduced here would represent a novel-controlled in vitro platform for cell–cell interactions^45,46^ and cell-drug interactions,^47^ overcoming some limitations of current techniques where the distribution of cells cannot be controlled, thus leading to inaccurate results.

The use of microfluidic fibers for drug-delivery applications have also been recently gaining momentum, with several studies featuring the impact of electrospinning fibers in the pharmaceutical industry.^7,8,48,49^ Microfluidic fibers offered distinct advantages over conventional dosage techniques (e.g., oral dosage), including high biocompatibility, effectiveness, high-aspect ratio, small diameter, and broad flexibility of fabrication.^7,8,48,49^ We speculate that fibers having equally spaced particles can further contribute to the improvement of such technologies, especially in relation to uniform dosage aimed at reducing adverse drug reactions.

The working principle employed here can also be used in conjunction with the one developed recently by Wang et al.^24^ The authors developed a solution to encapsulate cells in droplets in order to obtain fibers loaded with equally spaced droplets containing cells. However, the entire encapsulation process is stochastic, meaning that only around 30% of the particles will be encapsulated in a droplet, and there is no guarantee that consecutive droplets have cells in them. A potential solution to this problem was already found by others before, thanks to the particle ordering phenomenon introduced earlier in this manuscript. Briefly, equally spaced particles approach the encapsulation area with a constant frequency (at variance with the case when particles are not ordered), which is synchronized to the frequency of droplet formation (which is constant). By taking advantage of both the viscoelastic ordering^26,36,40^ and the controlled encapsulation phenomena^50,51^ in conjunction with the fiber formation presented here, we foresee the potential for the generation of other materials via the same principles. It is also worth mentioning that particle ordering and controlled encapsulation can also be achieved in purely inertial flow.^25,52,53^ However, the addition of polymer solutions has two strong advantages: (i) in viscoelastic ordering, particles are ordered on the channel centerline, while in the inertial ordering, they are generally ordered near the channel wall and on multiple lines^52^ and (ii) the addition of polymer solutions increase the elastic properties of the fibers, which is often desirable.

Future work should also investigate the possibility of fabricating fibers containing nonspherical particles. Currently, only very little has been done with respect to the ordering of nonspherical particles. To the best of our knowledge, there are only experiments performed to evaluate the transversal migration of spheroids in viscoelastic liquids (no ordering)^54^ and numerical simulations on the interactions between pairs of spheroids at different values of the aspect ratio.^33^ The numerical simulations^33^ suggests that the ordering of spheroid is possible; however, no evidence has been presented so far.

In summary, the fibers introduced here represent an important addition to the family of existing fibers. Their versatility is expected to open new research directions across several fields, including material science, chemistry, and biomedical engineering. We identified some areas where the viscoelastic ordering phenomenon can enhance existing manufactured fibers, while also leading to new instruments employable to address drug-delivery problems and also offering a new platform for the study of cell–cell interactions or cell-drug interactions.

## Conclusions

We here introduced a new class of materials featuring fibers loaded with equally spaced particles. The formation of equally spaced particles was facilitated by the recently discovered viscoelastic particle ordering phenomenon^26^ coupled to the well-established framework of fiber synthesis via ionic gelation.^10^ The experimental apparatus to manufacture the fibers was made of a simple syringe pump and of a long 100 μm tube exiting in a calcium chloride bath. The liquid suspending the particles was an aqueous solution of sodium alginate (NaA) and hyaluronic acid (HA). For a fixed HA concentration, we observed that fiber formation was stable within the range of concentrations investigated. We also observed that the volumetric flow rate and the spinning speed where two important parameters to tune the size of the fiber and the number of ordered particles along the fiber. Finally, we discussed the potential impact of these type of fibers across several fields, including material science, chemistry, biomedical engineering, and pharmaceutical research.

## Data Availability

The data underpinning the research are available upon reasonable request made to the corresponding author.

## Supporting Information Available

Formation of fibers with embedded particles in sodium alginate 1 wt % without hyaluronic acid at *Q* = 40 μL/min (MP4)
Formation of fibers with embedded particles in sodium alginate 0.75 wt % and hyaluronic acid 0.4 wt % at *Q* = 20 μL/min and spinning speed Ω_1_ = 110 rpm (MP4)
Formation of fibers with embedded particles in sodium alginate 0.75 wt % and hyaluronic acid 0.4 wt % at *Q* = 20 μL/min and spinning speed Ω_0_ = 0 rpm (MP4)
Formation of fibers with embedded particles in sodium alginate 0.5 wt % and hyaluronic acid 0.4 wt % at *Q* = 60 μL/min and spinning speed Ω_0_ = 0 rpm (MP4)
Formation of fibers with embedded particles in sodium alginate 0.5 wt % and hyaluronic acid 0.4 wt % at *Q* = 80 μL/min and spinning speed Ω_0_ = 0 rpm (MP4)
Formation of fibers with embedded particles in sodium alginate 0.75 wt % and hyaluronic acid 0.4 wt % at *Q* = 20 μL/min and spinning speed Ω_2_ = 167 rpm (MP4)
